# The diagnostic upper GI endoscopy camp: a pilot for enhancing service provision and training in eastern Uganda

**DOI:** 10.4314/ahs.v22i2.45

**Published:** 2022-06

**Authors:** Matthew Doe, Emmanuel Bua, Fred Bisso, Peter Olupot-Olupot

**Affiliations:** 1 Mbale Regional Referral Hospital, P.O. Box 921, Mbale, Uganda; 2 Mbale Clinical research Institute, www.mcri.ac.ug, P. O. Box 1966, Mbale Uganda; 3 Busitema University, Faculty of Health Sciences, P. O. Box 1460 Mbale, Uganda

**Keywords:** Gastrointestinal endoscopy, *Helicobacter pylori*, esophageal neoplasms

## Abstract

**Background:**

Upper gastrointestinal (UGI) symptoms are common in East Africa but there is limited diagnostic endoscopy availability. Surgical camps are a recognised method of providing intensive service provision and training. We describe a novel application of the camp model for diagnostic UGI endoscopy in eastern Uganda.

**Methods:**

A 7-day camp took place in an existing endoscopy department of Mbale Regional Referral Hospital. Patients with symptoms warranting investigation were invited for free diagnostic UGI endoscopy, biopsy and *H.pylori* testing.

**Results:**

148 patients underwent endoscopy. 47 were deemed to have significant pathology, 7 with malignancy. 61% had *H.Pylori*. A resident surgeon was trained and performed 55 supervised unassisted procedures.

**Conclusion:**

Our pilot has illustrated that camps are a safe and efficient way of providing intense endoscopy service and training in an established department. Camps can be utilised for scaling up much needed endoscopy services and training in low- and middle-income countries.

## Introduction

Upper gastrointestinal (UGI) symptoms warranting endoscopic evaluation are common in Uganda^1^. There is, however, limited availability of diagnostic UGI endoscopy in this region^1^,^2^,^3^ and patients are either treated empirically without formal diagnosis, or referred for services in specialisedcentres in the capital Kampala. Providing endoscopy services islabour intensive and expensive, requiring specialist equipment and experienced clinicians to be performed safely and effectively.

Surgical camps are a recognised method of providing intense surgical service and training. Camps typically involve volunteer clinicians and support staff performing a large number of free procedures in a short time period on a local patient population, normally at a location with limited existing surgical provision. Surgical service delivery in this manner has previously been found to be safe and effective in Uganda^4^,^5^,^6^. To date there are no published reports of endoscopy services and training in this manner worldwide.

Mbale Regional Referral Hospital (MRRH) is a government institution in eastern Uganda. In 2009, a diagnostic UGI endoscopy service was established through partnership between the hospital and Wales based organisation PONT (https://pont-mbale.org.uk). Services have run uninterrupted for 9 years, but with a low referral frequency - performing approximately 8 procedures per month. A camp providing free diagnostic UGI endoscopy was designed to boost capacity, increase local service awareness and train an additional endoscopist. We describe this camp - the first of its kind in the literature.

## Methods

For three weeks prior to the camp, advertisements were circulated via clinicians, community health workers, local radio, places of worship and posters. Patients over 18 years with symptoms of dyspepsia, dysphagia, haematemesis, vomiting or upper abdominal pain for more than two weeks were invited to call or attend the endoscopy department, describe their symptoms to a trained staff member and book a date where appropriate. Patients were prioritised according to age and symptoms.

The camp took place over a 7-day period during January and February 2019. Staff included one British volunteer general surgeon (joint advisory group (JAG)^7^ accredited), one resident surgeon (endoscopy novice), two experienced endoscopy nurses, a clinical officer, three additional nurses and one auxiliary.

The resident surgeon, clinical officer and three additional nurses were all new to the department - a full induction and orientation was provided prior to the camp start date for all staff who were new to the department.

The resident surgeon underwent training in diagnostic UGI endoscopy during the camp. The trainee was enrolled in a residency programme as a member of the College of Surgeons of East, Central and Southern Africa (the curriculum of which includes practice in UGI endscopy^8^) with MRRH as a base hospital. The resident committed to completing any remaining training after the camp and to providing an UGI endoscopy service in MRRH thereafter.

Prior to the camp start date, an objective setting meeting was performed between the resident surgeon trainee and the trainer (a JAG accredited volunteer surgeon). The resident was provided with appropraite learning materials several weeks in advance of the camp start date^9^,^10^.

Two donated Olympus endoscopy stack systems were used interchangeably. Patients were formally consented and had their findings explained in their first language where possible and were provided with health advice and/or prescription as per national management guidelines. All patients underwent *Helicobacter pylori* (*H.pylori*) rapidtest with gastric antral biopsies. Where necessary tissue biopsies were taken for histological analysis and patients invited back to a follow up appointment to receive results.

Data was collected prospectively using handwritten admission forms and procedure reports, then entered into an electronic MS-EXCEL database. Variables recorded included, but were not limited to; age, gender, indication, findings and H.pylori status.

Prior to the camp a quality assessment of the endoscopy department was conducted according to the pilot JAG Global Rating Scale. A resident surgeon was trained in UGI endoscopy during the camp, utilising JAG international Direct Observation of Procedural Skills forms as a framework for training and feedback^7^.

## Results

One-hundrend and fourty eight (148) patients underwent diagnostic UGI endoscopy (56 male, 92 female). Mean age was 50 years.

In Table 2, the indications (one recorded per patient) and corresponding findings (up to three recorded per patient) at endoscopy. Findings listed under ‘other’ are: Barrett's oesophagus, oesophageal dilatation, gastric and duodenal polyps, submucosal lesions, portal hypertensive gastropathy, gastric varices, bile acid reflux and duodenal diverticulum.

**Table 2:** Indications for Upper Gastrointestinal Endoscopy among Patients attending Camp

Findings/Indication	Epigastric pain (107/148)	Dysphagia (17/148)	Reflux (8/148)	Haematemesis (7/148)	Other (9/148)	Total
**Normal**	31	2	2	1	4	40
**Oesophagitis**	3	3	4	2	0	12
**Oesophageal** **candidiasis**	8	3	0	1	0	12
**Ca oesophagus**	0	5	0	1	0	6
**Hiatus hernia**	8	0	2	1	0	11
**Gastritis**	54	2	4	0	2	63
**Gastric ulcer**	4	1	0	0	0	5
**Ca stomach**	0	1	0	0	0	1
**Duodenitis**	10	1	0	0	0	11
**Duodenal Ulcer**	4	0	0	0	0	4
**Other**	9	9	2	2	3	25

Fourty-seven (47) patients were classed as having a significant diagnosis (malignancy, peptic ulceration, oesophageal or gastric varices, oesophagitis, oesophageal candidiasis or Barrett's oesophagus) - 7 of whom had a histologically proven malignancy or dysplasia (6 squamous cell carcinoma of the oesophagus, 1 gastric adenocarcinoma, 1 dysplastic gastric polyp) and were offered onward referral to specialist institutions in Kampala. Two cases of histologically proven Barrett's metaplasia of the oesophagus were identified.

61% (86/140) of those tested for *H.pylori* were positive. For the remaining 8 patients, either testing was deemed unsafe, or the antrum could not be accessed.

*Morbidity and Mortality:* There were no immediate complications of endoscopy. Two patients reattended the unit the day after their endoscopy; one suffered pleuritic chest pain which settled spontaneously and one complaining of worsening dysphagia (which was present pre-procedure) from an oesophageal malignancy who had been referred on to the Uganda Cancer Institute at the time of diagnosis. Four patients died within 30-days post-procedure, all of whom were found to have oesophageal or gastric malignancy. While none were able to afford formal diagnostic staging, their deaths were attributed to their underlying illness by clinicians in all cases.

*Cost:* The camp cost was 6,746,100 Ugandan Shillings ($1839) all of which was provided by international donations to the PONT charity. This donation covered the cost of all endoscopy procedures and histopathology processing for the duration of the camp. The only out-of-pocket expenses for the patient were for personal transport and any prescribed treatment.

*Training:* A local surgeon was trained over 50 hours of the camp (four full days and two half days) through short hands-on-competence building sessions. Basic UGI endoscopy competences were attained and the surgeon performed 55 successful procedures unassisted. The number of unassisted procedures successfully performed was found to increase each day of training with 5/14 (26%) on the first day and 7/8 (88%) on the final half day. An independent assessment of competence by clinicians at a separate established Ugandan endoscopy department has been arranged prior to the trainee starting independent practice.

## Discussion

The surgical camp model was safely and successfully replicated for diagnostic UGI endoscopy. Mobilisation proved effective with direct referral from a clinician or community health worker as the most successful methods.

## Limitations

The one-off method of training we describe here has both advantages and disadvantages. The resident surgeon did not formally achieve competence in diagnostic UGI endoscopy over the course of the camp, partly as they were unable to attain the evidence-based threshold of 200 procedures, as described by JAG and other international training programmes^11^. The resident has since pursued further endoscopy training in Kampala, however a follow-up system paired with provision of technology and materials, such as that described by Haglund et al^6^, is favoured for ongoing training.

Our patient cohort represents a small geographical and chronological snapshot with vulnerability to selection bias. Patients were not reimbursed for transport costs and thus were perhaps less likely to be co-morbid and or to have come from remote surrounding districts. Attendance of younger, better-connected patients is likely given the simple, short method of camp advertising.

With these limitations in mind, we can draw tentative conclusions on the burden of UGI pathology in eastern Uganda. There is a high incidence of squamous cell carcinoma of the oesophagus, in line with previous studies^1^,^2^,^7^. Hiatus hernia and *H.pylori*prevalence was found to be similar to previous African estimates^1^,^3^,^8^. This study represents the first reported cases of Barrett's metaplasia of the oesophagus in Uganda.

Intense endoscopy training programmes have previously been found to be successful^1^,^4^,^9^ and we feel that the camp provided a safe, familiar and effective learning environment for a local surgeon, with unique exposure to an impressive number of training cases and a wide range of pathology in a short space of time.

Scale up of diagnostic UGI endoscopy services in Uganda is urgently required in order to formally diagnose a range of important conditions that are currently treated empirically including peptic ulceration and oesophageal malignancy. We have described the first successful diagnostic UGI endoscopy camp of its kind.

While we recognise that camps are not a replacement of established healthcare services, we do feel that camps can be a safe and effective method of providing intensive service provision and training. Further camps of this kind should be considered to help scale up endoscopy services in low- and middle-income countries like Uganda.

## Figures and Tables

**Table 1:** Methods of Community Patient Mobilisation

Method of referral	Total
Doctor	30
Hospital or community health worker	30
Place of worship announcement	28
Poster	26
Radio announcement	15
Non government organisation	10
Inpatient (MRRH)	8
Social media	1

## Declarations

### Availability of data & materials

The datasets used and/or analysed during the current study are available from the corresponding author on reasonable request.
